# EGFRxCD16 bispecific antibodies orchestrate superior NK cell-mediated lysis of ovarian cancer and NSCLC cell lines in combination with oncolytic viruses

**DOI:** 10.1007/s00262-026-04470-4

**Published:** 2026-07-03

**Authors:** Sarah Gahbauer, Thomas Poiret

**Affiliations:** Department of Medicine Huddinge (MedH), Karolinska Institutet, Stockholm, Sweden

**Keywords:** NK cells, Oncolytic virus, Bispecific antibody, EGFR, CD16

## Abstract

Combination therapy integrating bispecific antibody (BsAb) and oncolytic viruses (OVs) to enhance antitumor immune response offers a promising therapeutic approach in comparison with OV treatment alone. This study aims to strengthen and characterize NK cell-mediated antitumor responses using modified oncolytic viruses, i.e., ONCOS-102 and ONCOS-204 genetically engineered to express Granulocyte-Macrophage Colony-Stimulating Factor (GM-CSF) and the ligand of inducible T-cell co-stimulator (ICOSL), respectively, in combination with a BsAb targeting CD16 and the epidermal growth factor receptor (EGFR). Changes in phenotype, degranulation, cytokine production, and cytotoxicity of NK cells induced by combined treatment were compared with those induced by individual treatment strategies against non-small cell lung cancer, malignant melanoma, and ovarian cancer. Using flow cytometry and colorimetry-based cytotoxic assays, our results showed that EGFRxCD16 BsAb was the main variable in driving NK cells toward an activated phenotype and enhanced NK function; while the OVs alone did not demonstrate drastic impact on NK cells. Interestingly, tumor preconditioning with OVs in combination with EGFRxCD16 BsAb showed the most potent NK cytotoxicity in comparison with EGFRxCD16 BsAb alone and appeared to synergize best against ovarian and EGFRmut lung tumor cell lines. Despite differences between tumor types, our data suggest a favorable interplay between OVs, especially ONCOS-102, and EGFRxCD16 BsAb  in sensitizing NK cell antitumor response in vitro. These results warrant further in vivo exploration and clinical translation of this promising combination of therapeutic modalities.

**Electronic supplementary material:**

The online version of this article (10.1007/s00262-026-04470-4) contains supplementary material, which is available to authorized users.

## Introduction

Advancements in genetic engineering have revitalized the field of oncolytic viruses (OVs), positioning them as a promising modality for anti-cancer therapy. To improve their oncolytic potential, several modified OVs have been developed and approved for clinical use, such as Talimogene laherparepvec (T-VEC) [1], Teserpaturev (G47Δ) [2], and Oncorine (H101) [3]. However, significant challenges remain, primarily concerning the timing between antitumor and antiviral immunity; rapid, pre-emptive viral clearance can compromise the therapeutic success of OVs before they exert an adequate antitumor effect [4]. To overcome some of these limitations, OVs were further modified to improve immune stimulatory properties and delivered in combination with other treatment modalities to fully leverage their immunomodulatory potential. These modifications include the incorporation of chemokine and cytokine transgenes (e.g., GM-CSF or IL-12) to promote immune cell infiltration, or the employment of various engager constructs to facilitate sustained cellular immunity [5]. As potent inducers of immunogenic cell death (ICD) [6], OVs can stimulate the release of tumor-associated antigens (TAAs) and danger-associated molecular patterns (DAMPs) [7, 8], thereby generating a local and potentially abscopal immune response accompanied by increased immune cell infiltration [9].

Natural Killer (NK) cells provide a vital first line of defense against virally infected and malignantly transformed cells; however, their antitumor function is often impaired in solid tumors due to inhibitory factors within the tumor microenvironment (TME) [10, 11]. To redirect NK cells against cancer, immune engagers such as CD16a (FcγRIIIA) bispecific antibodies (BsAb) allowed enhanced activation [12]: CD16 is a NK cell-activating receptor capable of triggering antibody-dependent cell-mediated cytotoxicity (ADCC) as well as chemokine and cytokine production by itself via engagement of CD16 with the Fc domain of an IgG antibody [13]. Given the rationale of targeting specific tumor antigens, the epidermal growth factor receptor (EGFR) has emerged as a particular compelling therapeutic target due to its overexpression on a broad range of cancers [14] and its role in promoting metastasis and tumor growth [15, 16]. The application of immunotherapy such as immune checkpoint blockage has been challenging especially in EGFR-mutated NSCLC due to its immunologically “cold” TME and high regulatory T-cell infiltration [17, 18]. However, NK cell-based therapies, including CAR-NK cells, their combination with OV therapy and bispecific/trispecific NK cell engagers demonstrated strong preclinical efficacy against EGFR-mutated cancers [19, 20]. NK cell function could significantly be improved by either modified OV treatment, several forms of NKCE constructs or the combination of both: This enhanced function manifests as increased antitumor cytotoxic potential, elevated cytokine production, robust NK proliferation as well as augmented infiltration capabilities into tumors in xenograft mouse models [21–23].

In this study, we investigated the combination of OV and BsAb directed NK cells by using two genetically modified chimeric oncolytic adenoviruses based on the same backbone (Ad5/3-D24): ONCOS-102 and ONCOS-204. ONCOS-102 (Ad5/3-D24.GM-CSF) expresses GM-CSF and ONCOS-204 (Ad5/3-D24-ICOS-L) expresses the co-stimulatory ligand Inducible T-Cell Costimulator Ligand (ICOS-L) in both soluble and surface-bound forms. We previously showed that both OVs and especially ONCOS-204 can potentiate T-cell activity in combination with EGFRxCD3 BsAb [24]. GM-CSF is the most common transgene for modified OVs (such as the FDA approved T-VEC for example), ICOS can be expressed to some level on activated NK cells [25] and is found at higher level in peripheral blood and tumor-infiltrating lymphocytes of patients with cervical cancer[26]. However, the role of OVs carrying GM-CSF and ICOS-L transgenes in modulating NK cells has not been investigated. Thus, we aimed to further explore the potential of therapeutic approaches that engage NK cells against EGFR-driven tumors, specifically investigating the impact of combining modified OVs (ONCOS-102/ONCOS-204) with an EGFR × CD16 BsAb on NK activation and solid tumor cell killing.

For this purpose, we performed detailed flow cytometry and cytotoxicity analyses to characterize NK cell responses in different conditions. We hypothesized that preemptively infecting tumor cells could enhance the function of EGFR × CD16 BsAb-activated NK cells, resulting in superior cytotoxicity against EGFR + tumor cell lines.

## Materials and methods

### EGFR + tumor cell lines and primary peripheral blood mononuclear cells

EGFR + ATCC cell lines A549 (CCL-185™), A375 (CRL-1619™), and SK-OV-3 (HTB-77 ™) were purchased from ATCC, and the H1975 (CRL-5908™) cell line was kindly given by Petra Hååg from Karolinska Institutet. A549 and A375 cells were cultured and expanded in DMEM (Cytiva); H1975 cells were cultured in RPMI-1640 (Cytiva) and SK-OV-3 cells in McCoy’s 5A medium (Sigma-Aldrich). All media were supplemented with 10% fetal bovine serum (FBS, Sigma-Aldrich) and 1% Penicillin/Streptomycin (Pen/Strep, Cytiva). All cell lines were tested for mycoplasma using the MycoAlert detection kit according to manufacturer’s instructions (Lonza). Peripheral blood mononuclear cells (PBMCs) were obtained from healthy donors and isolated using Ficoll-Hypaque density gradient (Cytiva). All cell lines and primary cells were cryopreserved in FBS supplemented with 10% dimethyl sulfoxide (Sigma-Aldrich) until use. All assays using SK-OV-3 ovarian cancer cells were performed using PBMCs isolated from female donors.

### EGFR expression in A549, H1975, A375, and SK-OV-3 cell lines

EGFR expression of tumor cell lines was assessed after viability staining using 0.5 µL LIVE/DEAD™ Fixable Aqua Dead Cell Stain Kit (Molecular Probes, Life technologies) followed by anti-EGFR PE (BD). To account for differences in autofluorescence between cell lines, the geometric mean fluorescence intensity (gMFI) was used to define EGFR expression, quantified as the fold change of stained gMFI over unstained gMFI.

$$\left( {\frac{{MFI_{stained} }}{{MFI_{unstained} }}} \right)$$

### EGFRxCD16 BsAb binding to EGFR + tumor cells and CD16 + NK cells

The recombinant anti-CD16xAnti-EGFR (EGFR × CD16) BsAb is composed of a single-chain variable fragment domain (scFv) targeting EGFR and a crystallizable fragment (Fc) derived from CD16 domain (custom made by Creative Biolabs, Shirley, NY, USA). For the binding of the EGFRxCD16 BsAb with surface EGFR, 0,25e6 EGFR + (SK-OV-3, A375, A549, and H1975) and EGFR- (Kasumi-1) tumor cells were washed with PBS and incubated 20 min at 4 °C with 250 ng/ml EGFRxCD16 BsAb. After a wash, cells were then stained using 0.25 µL LIVE/DEAD™ Fixable Aqua Dead Cell Stain and anti-IgG Fc PE (BioLegend, clone M1310G05) for 30 min at room temperature in the dark. To assess the binding of the EGFRxCD16 BsAb with surface CD16, 0.5e6 PBMCs from female donors (n = 3) were incubated with EGFRxCD16 BsAb as described above followed by an antibody cocktail consisting of LIVE/DEAD™ Fixable Aqua Dead Cell, anti-CD3, anti-CD56, and anti-IgG Fc for 30 min at room temperature in the dark (**Suppl Table 1a**).

### Titration of EGFRxCD16 BsAb by NK cell activation

For BsAb titration, tumor cells were seeded at 25 K/well in a 24-well plate (Falcon). After 24 h, PBMCs were added at effector:target (E:T) ratio of 10:1 together with different concentrations of EGFRxCD16 BsAb ranging from 0 to 10,000 ng/ml. Following 4 h co-incubation, PBMCs were collected, stained for viability and the surface markers CD3, CD56, and CD107a as listed in **Suppl. Table 1**.

### Modified Oncolytic Viruses and ICOS-L and GM-CSF expression in A549 and H1975 cancer cell lines

The oncolytic adenoviruses (OVs) were provided by Circio Holding ASA (Oslo, Norway). ONCOS-102 (produced by Biovian, Finland) which induces GM-CSF expression, ONCOS-204 (produced by OD260 Inc., USA), which induces ICOS-L expression and control vehicle virus (AdV5/3-D24-E3) were used as previously described [24]. OV stocks were kept at -80 °C until use. Transgene expression induced by ONCOS-102 and -204 in tumor cells was assessed as previously reported using a human GM-CSF ELISA kit (Mabtech, AB 3480-1H-6) and A647 anti-ICOSL antibody (clone 2D3/B7-H2, BD), respectively [24].

### NK cell phenotype, degranulation, and cytokine production assays

In a 24-well plate (Falcon), 50 k tumor cells were incubated overnight prior to addition of 100 viral particles/cell (MOI 100) of the different OVs. After 24 h, the medium was removed, and PBMCs were added at a 10:1 (E:T) ratio in NK MACs medium supplemented with 1% (v/v) Pen/Strep and 10% FBS.

For degranulation and cytokine production analysis, EGFRxCD16 BsAb was added at suboptimal concentration of 250 ng/mL together with 10 µg/mL Brefeldin A (Sigma-Aldrich), Golgi stop (BD Biosciences), and anti-CD107a antibody PE (BD). After 4 h co-incubation, PBMCs were collected, stained for viability followed by surface antibodies in Horizon™ Brilliant Stain buffer (BD). Cells were then fixed and permeabilized (Foxp3 Transcription Factor Staining Buffer, Thermo Fisher) before intracellular staining. A list of antibodies is found in **Suppl Table 1a**.

For phenotype analysis, PBMCs were collected after 24 h co-incubation and stained for viability and surface antibodies listed in **Suppl Table 1b**.

### NK sorting and cytotoxicity assay

Human NK cells were isolated from healthy donor-derived PBMCs using the MACS NK cell Isolation Kit (Miltenyi Biotec) according to the manufacturer’s instructions. Isolation purity was assessed using flow cytometry targeting purity > 90% of live CD3neg cells. Cytotoxic assays were performed by seeding 4 k cells/well in 96-well flat bottom plates (TPP) overnight. Different OVs were then added at a MOI of 100 for 24 h. Following infection, the medium was replaced, and the co-culture was set with the addition of 12 k sorted NK cells (E:T ratio 3:1) and 250 ng/ml BsAb in NK MACs medium supplemented with 1% Pen/Strep and 10% FBS. After 24 h, the medium was removed, and the wells were gently washed with PBS before the addition of 100 µL RPMI-1640 medium supplemented with 10% FBS and 10 µL WST-1 Cell Proliferation reagent (Roche). After 1 h incubation at 37 °C, absorbance was measured between 420 and 450 nm (according to the cell line maximum absorption following manufacturer instruction) using a microplate reader (BMG Labtech). Specific killing for each condition was calculated based on absorbance (Abs) as follows and specifically described in concerned figures:

$${\text{Specific killing}} \left( \% \right) = 100 \times \left( {1 - \frac{{{\text{Abs specific condition}}}}{Abs NK only}} \right)$$

### Data analysis and statistics

Flow cytometry analysis was performed using CytoFLEX (Beckman Coulter). Data were analyzed using FlowJo V.10.10.0 (BD Life Sciences). Statistical analyses were performed using GraphPad Prism 10 (GraphPad Software, San Diego, CA, USA). Paired samples exposed to two or more different conditions (BsAb or OV presence) were analyzed using non-parametric Wilcoxon signed-rank test and Friedman test with Dunn’s correction, respectively. The comparison of OV conditions between different cell lines was analyzed using Kruskal–Wallis test. Significance was defined as α < 0.05.

## Results

### Validation of OVs and selection of a suboptimal EGFRxCD16 BsAb concentration

**Fig. 1:**
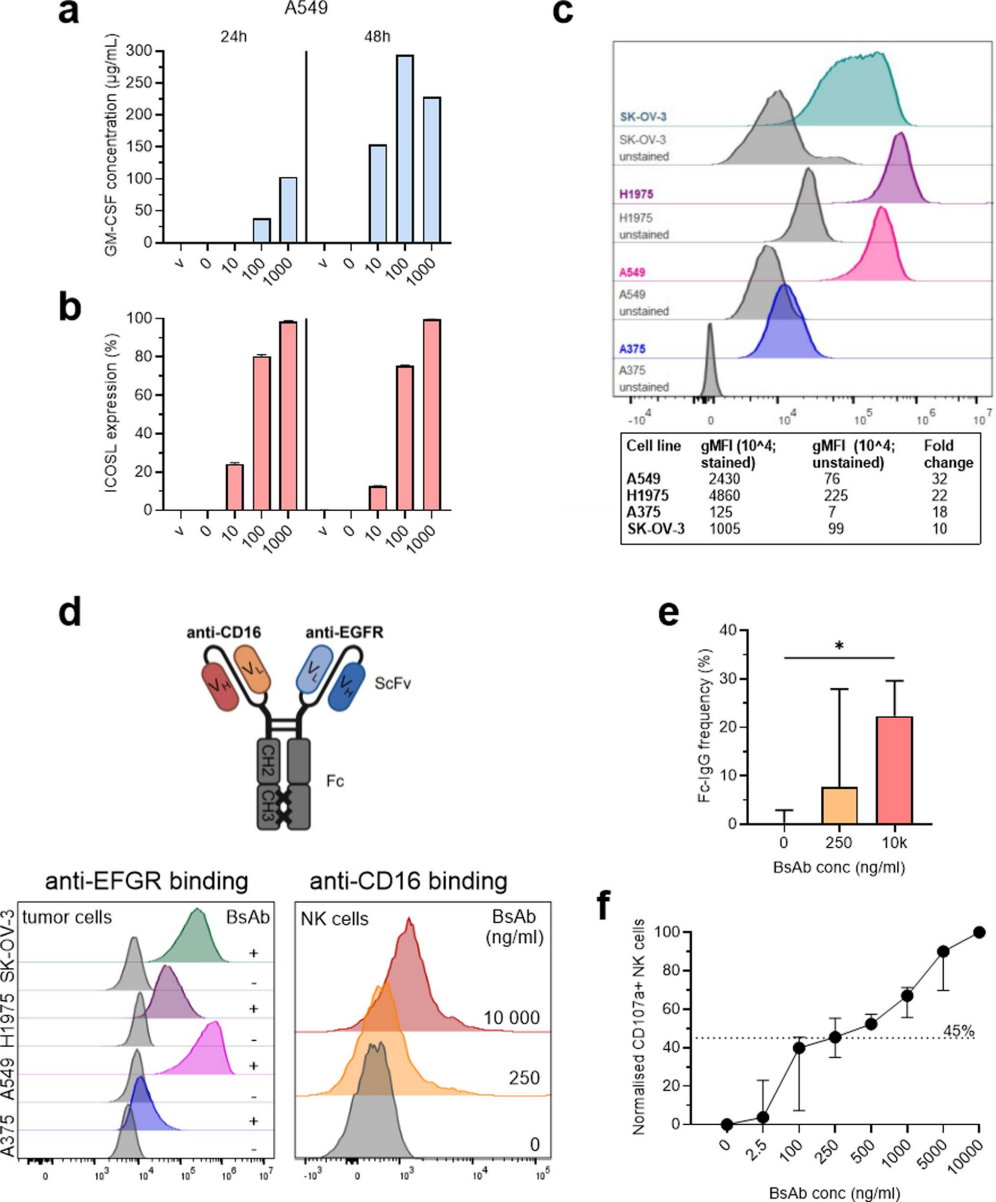
Characterization of tumor cell lines and titration of EGFRxCD16 BsAb and OVs. Production of GM-CSF (**a**) and surface expression of ICOS-L (**b**) by A549 tumor cells induced by different MOI (0, 10, 100, and 1000) of ONCOS-102 and ONCOS-204, respectively. **c.** EGFR expression in SK-OV-3, H1975, A549, and A375 cancer cell lines**. d.** Schematic of the custom-made EGFRxCD16 BsAb (inspired from Creative Biolabs) and histogram of its binding to EGFR + tumor cell lines (left) and CD3-CD56 + NK cells (right). **e.** Frequency of Fc-IgG bound to CD3-CD56 + NK cells (N = 3) using different concentrations of EGFRxCD16 BsAb. **f.** Titration of EGFRxCD16 BsAb on NK cells by normalized expression of CD107a. The Friedman test with Dunn’s correction was used to compare between the three different BsAb concentrations (**e**). * p < 0.05

The potential of OVs to express their respective transgenes was assessed in the A549 cell line at different MOIs, using the vehicle as control (Fig. 1a-b): 24 h after infection with OVs, a significant level of GM-CSF was observed at MOI 100 and ICOSL expression at MOI 10. After 48 h, a MOI 100 of ONCOS-102 and ONCOS-204 induced the secretion of GM-CSF above 100 µg/ml and expression of ICOS-L above 75% by A549 tumor cells. The induction of transgene expression by ONCOS-102 and ONCOS-204 was further confirmed in H1975 cells (**Suppl Fig. 1a**). Surface EGFR expression on different tumor cell lines was then compared (Fig. 1c): Lung tumor cells lines, (i.e., H1975 and A549 cells) exhibited the highest expression level (gMFI > 2000) of EGFR while the ovarian cancer cell line SK-OV-3 showed the largest spread of EGFR expression and the lowest expression in proportion to the control.

To validate the custom-made EGFRxCD16 BsAb, its binding to surface EGFR + on tumor cells and CD16 + on NK cells was demonstrated by indirect staining of the anti-IgG Fc present on the EGFRxCD16 BsAb (Fig. 1d-e**, Suppl Fig. 1b**). To assess the functional impact of OVs and associated transgenes on NK cells when combining OVs and EGFRxCD16 BsAb, a suboptimal concentration of 250 ng/ml BsAb was determined to correspond to a 40–60% increase in degranulation, characterized by CD107a frequency in NK cells (Fig. 1f**, Suppl Fig. 1c**). The degranulation of NK cells was then evaluated at different BsAb concentrations when co-cultured with the EGFR-mutated tumor cell line H1975 to evaluate the possible use of the EGFRxCD16 BsAb. NK cells showed a significantly higher frequency of CD107a + cells (median > 15%, p < 0.05) when supplemented with 250 ng/ml in comparison with the controls (**Suppl Fig. 1d**).

Altogether, ONCOS-102 and -204 efficiently induced expression of their respective transgenes and EGFRxCD16 BsAb significantly increased NK cell degranulation.

### EGFRxCD16 BsAb-induced activation phenotype and increased frequency of degranulating and cytokine-producing NK cells

**Fig. 2:**
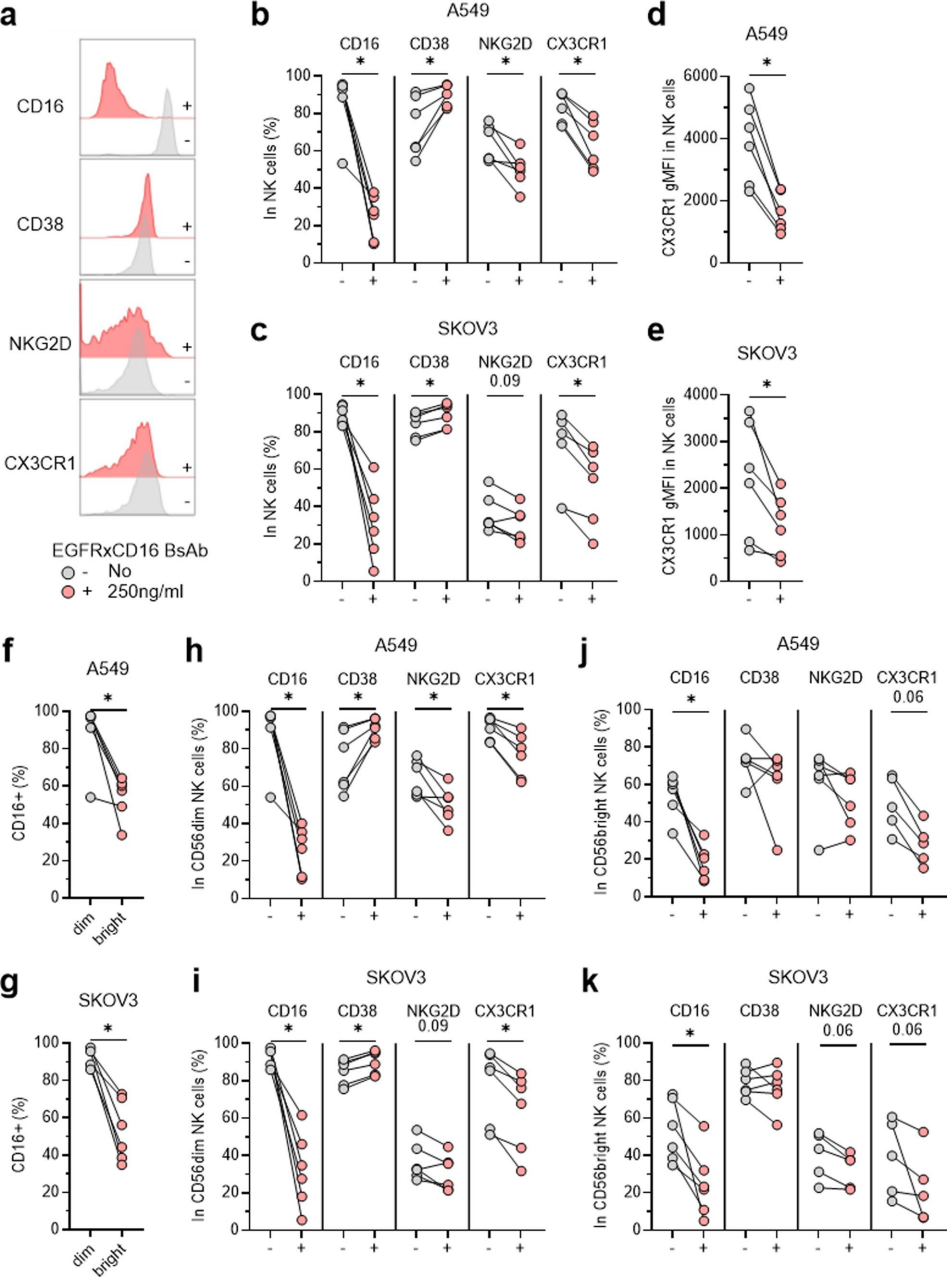
NK cell phenotypic changes induced by co-culture with tumor cells and EGFRxCD16 BsAb. **a.** Representative flow cytometry of CD16, CD38, NKG2D, and CX3CR1 expression in CD3-CD56 + NK cells. Analysis of changes in (**b-c**) frequency of CD16, CD38, NKG2D, and CX3CR1 NK cells and (**d-e**) expression of CX3CR1 NK cells induced by 250 ng/ml EGFRxCD16 BsAb after 24 h co-culture with (**a, b, d**) A549 cells and (**c & e**) SK-OV-3 cells. Comparison of different frequencies of CD16 + NK cells in CD56dim and CD56bright NK cells after co-culture with A549 (**f**) and SK-OV-3 (**g**). Analysis of frequency changes of CD16, CD38, NKG2D, and CX3CR1 CD56dim (**h & i**) and CD56bright (**j & k**) NK cells induced by 250 ng/ml EGFRxCD16 BsAb after 24 h co-culture with A549 cells (**h & j**) and SK-OV-3 cells (i **& k**). N = 6 individuals. The Wilcoxon test was used to compare the two conditions and subsets. * p < 0.05

Phenotypic changes were evaluated on NK cells after a 24 h co-culture with four different EGFR + tumor cell lines to characterize the impact of the EGFRxCD16 BsAb (**Suppl Table 1a, Suppl Fig. 1e**). When co-cultured with tumor cells, apart from increased CD161 + CD56dim cell frequency, the addition of the BsAb did not have a significant impact on the proportion of CD57, NKp46, and TIGIT-expressing NK cells (**Suppl Fig. 2a-c**). However, supplementation of BsAb significantly decreased the frequency of CD16 + and NKG2D + NK cells while increasing the frequency of CD38 + NK cells (p < 0.05, Fig. 2a-c**, Suppl Fig. 2d-e**). We also observed a reduced frequency and surface expression of CX3CR1 on NK cells (p < 0.05, Fig. 2a-e). Since CD56dim exhibited a higher frequency of CD16 than CD56bright cells (p < 0.05, Fig. 2f-g), we then investigated the phenotypic changes induced by the EGFRxCD16 BsAb within these two NK subpopulations: As expected, while both subpopulations saw their CD16 + frequency reduced by the addition of the BsAb, the CD56dim NK cell phenotype (Fig. 2h-i) changed more significantly than the CD56bright cell subset (Fig. 2j-k) with a decreased frequency of NKG2D + , CX3CR1 + , and increased frequency of CD38 + NK cells. To a lower extent, we also observed an increased frequency of CD161 + and TIGIT + CD56dim NK cells upon addition of the BsAb (**Suppl Fig. 2f-g**).

**Fig. 3:**
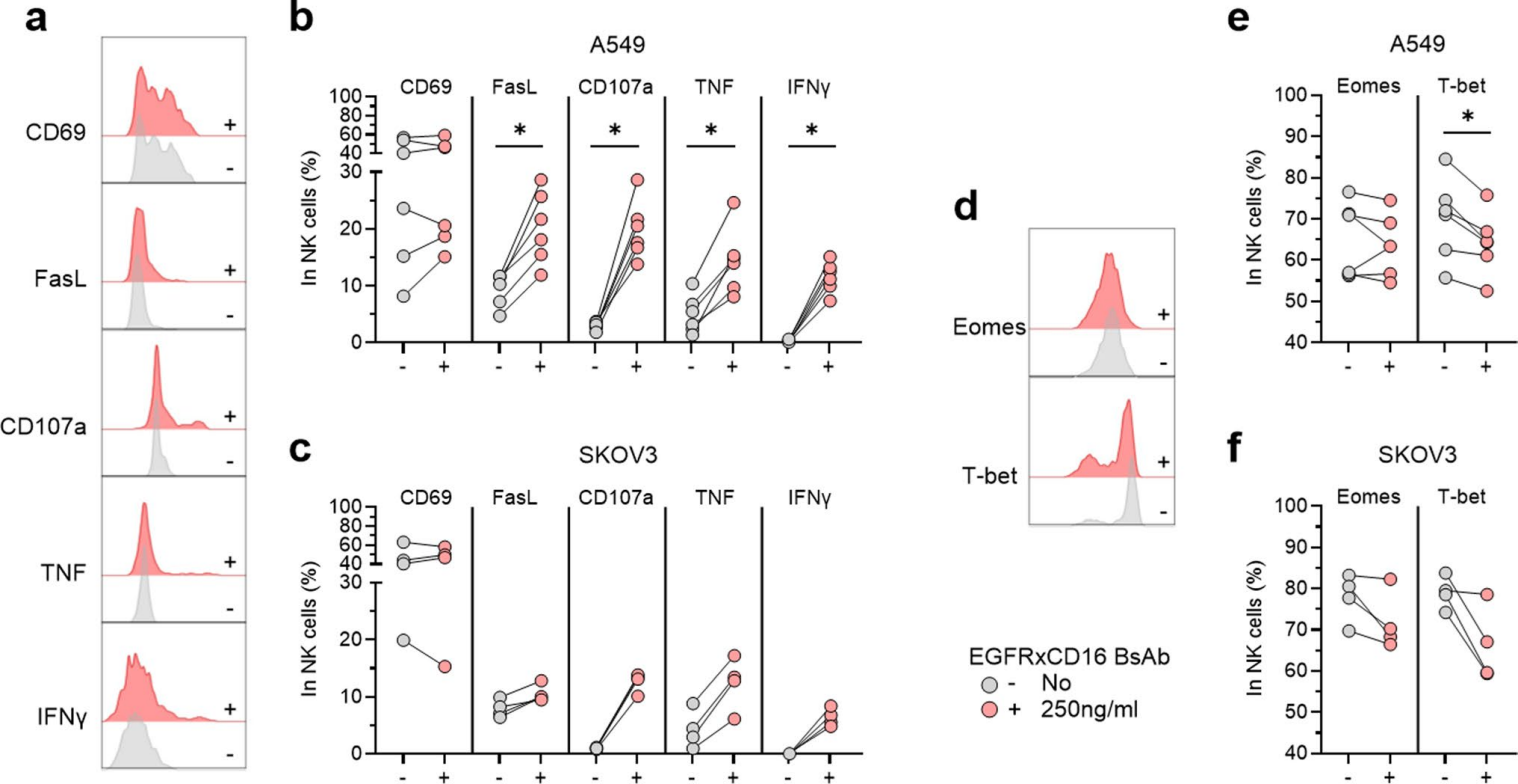
EGFRxCD16 BsAb increased NK cell function**. a.** Representative flow cytometry of CD69, FasL, CD107a, TNF, and IFNγ expression in CD3-CD56 + NK cells. Increased functionality of NK cells induced by EGFRxCD16 BsAb after 4 h co-culture with (**b**) A549 cells and (**c**) SK-OV-3 cells. Representative histogram (**d**) and frequency (**e–f**) of Eomes and T-bet in CD3-CD56 + NK cells induced by EGFRxCD16 BsAb after 4 h co-culture with (**e**) A549 cells and (**f**) SK-OV-3 cells. **b & e** N = 6, and **c & f** N = 4 individuals. The Wilcoxon test was used to compare the two conditions. * p < 0.05

Following these observations, we next investigated how 4 h exposure with EGFRxCD16 BsAb influences the function of NK cells toward the different tumor cell lines (**Suppl Table 1b, Suppl Fig. 1f**): The addition of the BsAb did not increase the frequency of CD69 + NK cells; however, in addition to the known increased frequency of CD107a + NK cells, the BsAb also induced a significantly increased proportion of FasL + and cytokine (TNF and IFNγ)-producing NK cells (p < 0.05, Fig. 3a-c**, Suppl Fig. 3a-b**). Transcription factor changes were also caused by the BsAb, resulting in a decreased frequency of T-bet + NK cells, especially when co-cultured with A549 tumor cells (Fig. 3d-f**, Suppl Fig. 3c-d**). While most NK cells expressed Eomes, no significant change in its expression was induced by the BsAb (Fig. 3d-f). Thus, Eomes + T-bet + co-expressing cells represented the highest proportion of NK cells and the addition of BsAb tended to decrease this proportion in favor of Eomes-T-bet- NK cells (p < 0.05, **Suppl Fig. 3E-F**).

Therefore, EGFRxCD16 BsAb efficiently induced an activated phenotype of NK cells, especially in the CD56dim subpopulation and significantly increased the frequency of degranulating and cytokine-producing NK cells against different tumor cell lines.

### OV-infected tumor cells alone induced minor changes in NK cells.

**Fig. 4:**
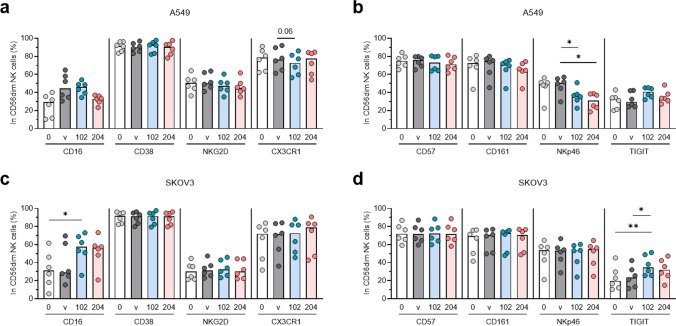
OV-infected tumor cells induced minor phenotypic changes without affecting the function of EGFRxCD16 BsAb stimulated CD56dim NK cells. Analysis of phenotypic changes in CD56dim NK cells induced by 24 h co-culture with EGFRxCD16 BsAb and OV-infected (**a & d**) A549 and (**b & e**) SK-OV-3 cells. **c.** Analysis of changes in CX3CR1 expression in CD56dim NK cells induced by different OV-infected tumor cells. Comparison of function of NK cells exposed to different OV-infected (**f**) A549 and (**g**) SK-OV-3 cells. Comparison of Eomes and T-bet expressions in NK cells exposed to different OV-infected (**h**) A549 and (**i**) SK-OV-3 cells. N = 6 individuals. The Friedman test with Dunn’s correction was used to compare between the four different conditions. * p < 0.05 and ** p < 0.01

Next, we aimed to evaluate how EGFR + tumor cells infected solely with modified OVs, such as ONCOS-102 and ONCOS-204, may influence the phenotype and function of NK cells. While no activation pattern was observed on NK cells following their co-culture with tumor cells (Fig. 4a-e**, Suppl Fig. 4**), a few differences were noted: First, CD56dim NK cell CX3CR1 frequency and expression were significantly reduced when co-cultured with ONCOS-102-infected A549 tumor cells in comparison with controls (Fig. 4a). This was observed neither in the CD56bright subset (**Suppl Fig. 4a-c**), nor in other tumor cell lines (Fig. 4b**, Suppl Fig. 4f-h**). A549 infected with ONCOS-102-induced other minor phenotypic changes, such as a reduced frequency of NKG2D + in CD56dim and CD56bright NK cells (Fig. 4a**, Suppl Fig. 4a**), while infection with vehicle adenovirus led to an elevation in TIGIT + NK cell frequency (Fig. 4d**, Suppl Fig. 4d**). NK cells co-cultured with SK-OV-3 cells showed only an increased frequency of CD161 + and CD57 + CD56dim cells when tumors cells infected with ONCOS-204 and ONCOS-102, respectively, in comparison with the controls (Fig. 4e**, Suppl Fig. 4e**). After a 4-h co-culture with OV-infected tumor cells, NK cells did not show signs of improved or decreased activation (Fig. 4f-g**, Suppl Fig. 5a-b**) or changes in their transcription profile (Fig. 4h-i**, Suppl Fig. 5c-d**).

Therefore, in our setup, tumors infected by OVs alone caused only minor phenotypic changes without affecting the frequency of degranulating and cytokine-producing NK cells either positively or negatively at the measured timepoints.

### GM-CSF and ICOS-L expression induced by OVs did not cause drastic phenotypical and functional changes of NK cells when combined with EGFRxCD16 BsAb.

**Fig. 5:**
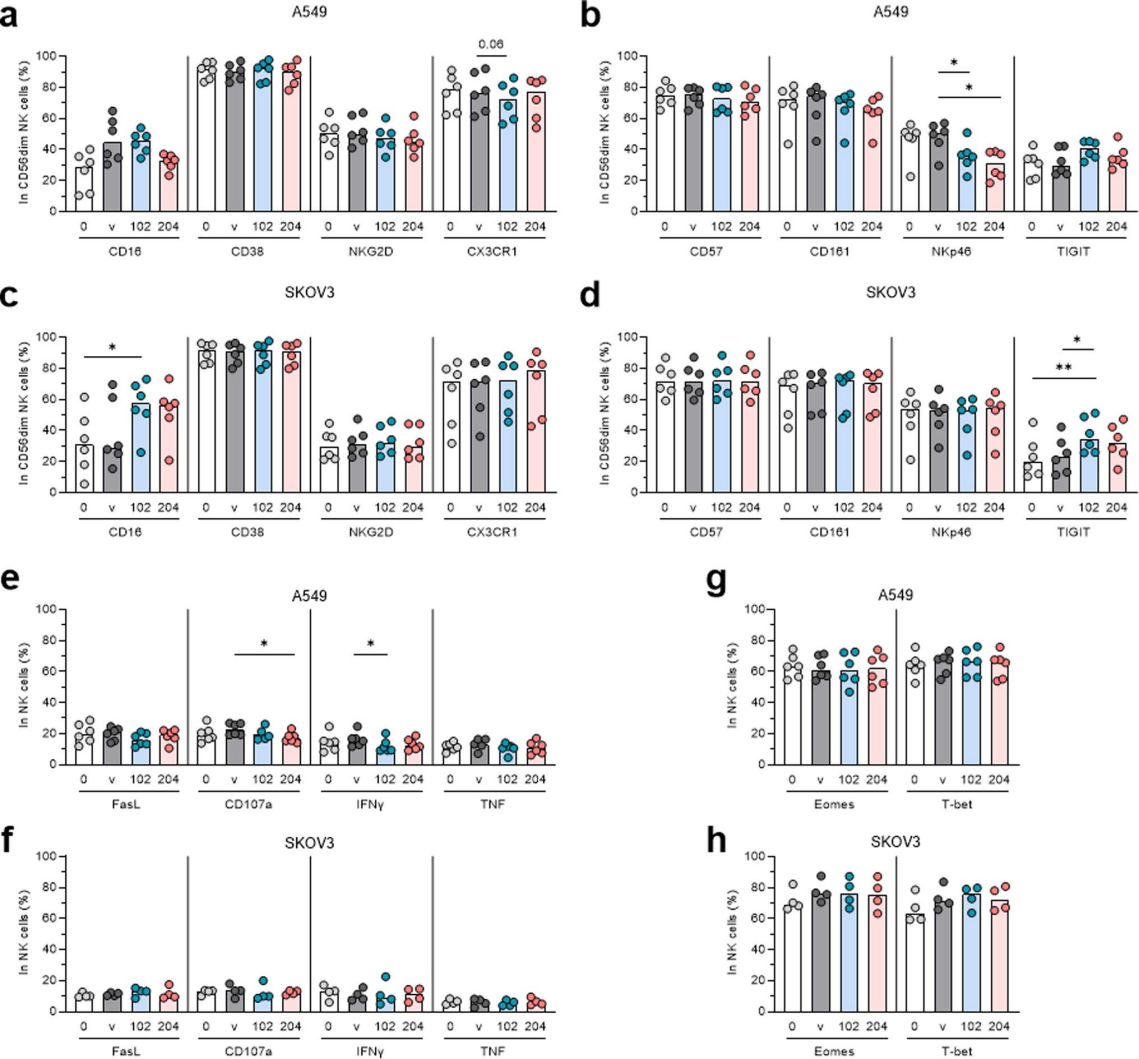
Conditioning of tumor cells with OVs caused minor functional changes in NK cells stimulated with EGFRxCD16 BsAb. Analysis of phenotypic changes in CD56dim NK cells induced by EGFRxCD16 BsAb during 24 h co-culture with OV-infected (**a-b**) A549 and (**c-d**) SK-OV-3 cells. Functionality of NK cells induced by EGFRxCD16 BsAb after 4 h co-culture with OV-infected (**e–f**) tumor cells. Transcription factor expression after 4 h co-culture with EGFRxCD16 BsAb and OV-infected (**g-h**) tumor cells. N = 6 individuals. The Friedman test with Dunn’s correction was used to compare between the four different conditions. * p < 0.05 and ** p < 0.01

We showed above that the EGFRxCD16 BsAb caused a generally increased activated phenotype and improved NK function of NK cells (Figs. 2 & 3); thus, we further investigated the potential additional effect of OV-infected tumor cells on the activated NK cells. Similarly to what was observed when OVs were used alone (Fig. 4), NK cells also activated by the EGFRxCD16 BsAb induced specific changes in their NK phenotype (Fig. 5a-d**, Suppl Fig. 6a-b**): A549 infected with modified OVs led to a decrease in NKp46 frequency (Fig. 5b) and ONCOS-102-infected SK-OV-3 cells induced an increased frequency of CD16 + and TIGIT + CD56dim NK cells **(**Fig. 5c-d) in comparison with the controls. Similarly, a slight increase of CD107a + and IFNγ + NK cells frequencies was noted when exposed to vehicle OV-infected A549 cells in comparison with exposure to the modified OVs (Fig. 5e). No other significant changes in monitored functions induced by OVs were observed in different cell lines (Fig. 5f-h**, Suppl Fig. 6c-f**).

Overall, EGFRxCD16-activated NK cells did not show improved or reduced degranulation and cytokine production upon exposure to modified OV-infected tumor cells in our setup and time of measurement.

### *Combination of EGFRxCD16 BsAb and OVs significantly improved killing of EGFR* + *tumor lines*

**Fig. 6:**
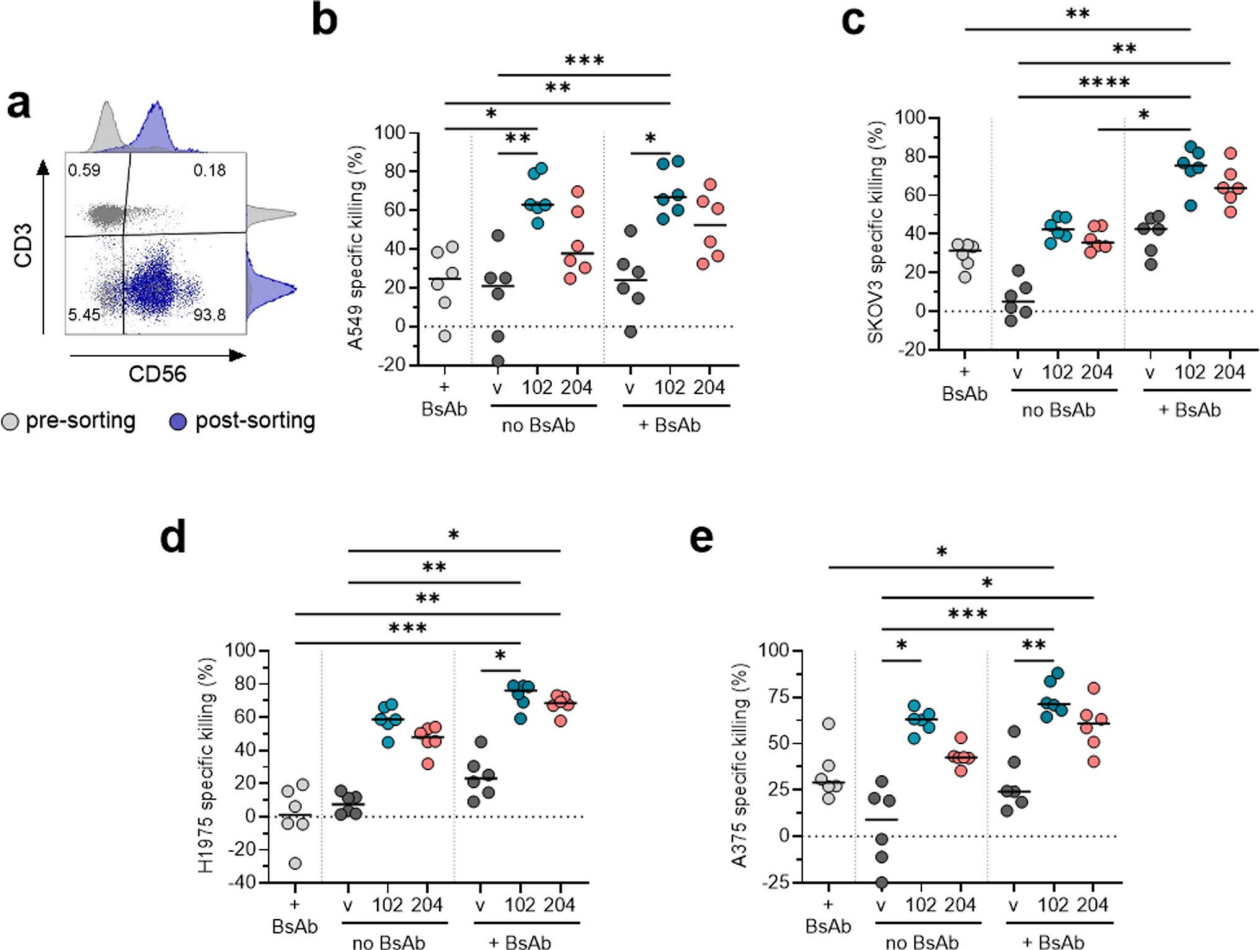
Combination of EGFRxCD16 BsAb and ONCOS-102 induced superior killing of tumor cells. **a.** Representative flow cytometry of NK cell proportion pre- and post-sorting. Comparison of percentage of specific (**b**) A549, (**c**) SK-OV-3, (**d**) H1975, and (**e**) A375 killing between different conditions (i.e., between OVs and with/without EGFRxCD16 BsAb tumor cell lines) using the formula. N = 6 individuals. The Friedman test with Dunn’s correction was used to compare between the four different conditions. * p < 0.05; ** p < 0.01; and *** p < 0.001

Following the characterization of the effect of EGFRxCD16 BsAb alone and OVs alone on the phenotype and function of NK cells, we investigated their impact on the cytotoxicity of NK cells. After NK cell enrichment (Fig. 6a), the cells were co-cultured for 24 h with EGFR + tumor cells in different conditions. In line with the earlier observation, the addition of the EGFRxCD16 BsAb alone significantly decreased the viability of all tumor cells, with the exception of H1975 tumor cells, as demonstrated by reduced absorbance (**Suppl Fig. 7a**). Accordingly, when co-cultured with NK cells, the EGFRxCD16 BsAb-specific killing of H1975 was minimal compared to the efficient killing observed against A375 and SK-OV-3 cells (**Suppl Fig. 7b**). We next compared the viability of tumor cells infected with different OVs and co-cultured them with sorted NK cells: ONCOS-102-infected tumor cells showed the consistent lowest viability across the different cell lines (p < 0.01, **Suppl Fig. 7c**). This translated to higher virus-specific killing of ONCOS-102 in comparison with the vehicle virus regardless of the absence (p < 0.01, **Suppl Fig. 7d**) or the presence (p < 0.01, **Suppl Fig. 7e**) of the EGFRxCD16 BsAb in the co-culture. When normalized to the baseline (tumor cells co-cultured with sorted NK alone, see M&M), the combination of EGFRxCD16 BsAb and modified OVs—particularly ONCOS-102—yielded the greatest reduction in tumor viability compared to EGFRxCD16 BsAb alone (p < 0.01, Fig. 6b-e). Interestingly, while the combination advantage seems minor in some cell lines, for instance, A549 cells where tumor viability with or without BsAb is not significant (around 60% in ONCOS-102 condition ± BsAb, Fig. 6b), the combination of OVs and EGFRxCD16 BsAb showed a clear additive effect on the viability of SK-OV-3 and H1975 tumor cell lines (Fig. 6c-d).

In conclusion, the combination of EGFRxCD16 BsAb and modified OVs improved the sensitization of several tumor cell lines to NK cell activity. The combination showed improved killing of SK-OV-3 ovarian cancer tumor cells and especially H1975 NSCLC tumor cells, which did not benefit from EGFRxCD16 BsAb exposure alone.

## Discussion

Cancer treatment strategies have been reshaped by advances in immunotherapy. Notably, NK-mediated therapy has demonstrated significant potential due to the cells’ enhanced cytotoxic capacity, their MHC-independent recognition, and the excellent safety profile observed during clinical trials. To augment NK function and achieve maximum antitumor effects, NK cell engagers were developed; however, multiple immunosuppressive mechanisms within the tumor microenvironment (TME) have limited their application in solid tumors. Here, we demonstrated that infecting several tumor cell lines with different modified oncolytic adenoviruses is an attractive approach to prime the tumor for NK cell-engaging BsAb-based therapy. We report that despite limited changes in the NK phenotype and function induced by preconditioning tumor cells with OVs, NK cells show enhanced killing when both therapies are combined.

Consistent with the previous studies [27–29], the addition of BsAb triggered degranulation, cytokine release, and cytotoxicity against EGFR + tumor cells by engaging CD16, which is recognized as the most potent activating receptor on NK cells [30]. Interestingly, BsAb treatment also induced a significant shift in NK cell phenotype, characterized first by a reduced frequency of CD16 + cells. This reduction may be attributed to CD16 shedding from the NK cell surface following BsAb-mediated target cell stimulation. This process mediated by metalloproteases [31] was reported to facilitate NK cell detachment from targets after successful lysis, thereby promoting serial killing by disassembling the immunological synapse [32]. While this mechanism provides a plausible explanation for the observed loss of CD16 expression, we cannot exclude the possibility of steric hindrance between the EGFRxCD16 BsAb and the anti-CD16 mAb clone used for flow cytometry analysis. However, because CD38 and CD16 are functionally dependent using common signaling machinery when activated [33, 34], the observed increased CD38 + NK cell frequency suggests that EGFRxCD16 BsAb successfully activated NK cells via CD16 receptor engagement. Treatment with the EGFRxCD16 BsAb also resulted in a reduced frequency of NKG2D + NK cells and a decrease in CX3CR1 expression: Exposure to NKG2D ligands can induce receptor downregulation and impaired NK cell responsiveness, partly through upregulation of inhibitory receptors like TIGIT in competition with DNAM-1 [35]. In our study, the BsAb used in this study did not engage the NKG2D receptor, but we cannot exclude a possible interaction with MICA/B expression on the target cells that would cause this reduced NKG2D frequency. Nevertheless, as we did not find significant upregulation of TIGIT, nor a suppressed function, this mechanism might not be the main driver of the observed reduction in NKG2D + NK frequency. The CX3CR1 downmodulation may suggest a reprogramming of NK trafficking and maturation by BsAb, as has been shown in response to CD16 engagement [36] and pro-inflammatory cytokines [37] suggesting a possible contribution to the polarization to Th1 responses.

We also demonstrated that the phenotypical changes appeared more pronounced within the CD56dim NK population, due to the higher inherent CD16 expression in this population than in CD56bright NK cells. As CD56dim NK cells are considered more mature with potent cytolytic activity and ADCC function that participate in antitumor responses [38], a specific stimulation of this subset may be beneficial for NK cell-based immunotherapy. Yet, it would be valuable to investigate the BsAb effect on cytokine-induced memory-like NK cells derived from the CD56bright NK subset that have recently shown to have superior therapeutic potential compared to conventional NK cell product [39].

Preconditioning tumor cells with various OVs (AdV5/3-D24-E3-based) was shown to enhance T-cell function and T-cell-mediated cytotoxicity [24]. However, these OV transgenes, designed primarily for T cells, seem non-ideal for NK cells, which express very low ICOS in their resting state [25] and lack a GM-CSF receptor altogether [40]. Yet, the ICOS-ICOS-L interaction showed enhanced cytotoxicity and IFN-γ production by NK cells [25], it would be relevant to study the impact of ONCOS-204 on activated/expanded NK cells. Only minor changes were observed on NK cell phenotype and function after OV preconditioning, however, in an in vivo setup, expression of GM-CSF may modulate the phenotype and functionality of other immune cells, such as dendritic cells, monocytes, and macrophages, to potentiate NK function via activating cytokines (e.g., IL-12, IL-15, and IFNs) [41, 42]. Nevertheless, we show that NK cytotoxic cells are modulated by both viruses, potentially sensing endoplasmic reticulum and cellular stress in NSCLC through NKp46 and NKG2D [10, 43], as well as influence trafficking potential through the CX3CR1 pathway.

Interestingly, while the preconditioning with the different OVs before co-culture of NK cells with or without EGFRxCD16 BsAb did not show drastic improved function of NK, the OV-BsAb combination demonstrated increased tumor-specific killing with the BsAb-ONCOS-102 combination exhibiting the highest killing enhancement suggesting a possible tumor cell sensitization by the OVs. Furthermore, we observed that H1975 cells displayed the highest surface EGFR (gMFI) levels; however, they were the least susceptible to BsAb-specific killing compared with the other tumor cell lines. This could be due to i) the use of a suboptimal concentration of EGFRxCD16 BsAb or ii) their specific EGFR mutations (T790M and L858R, in exons 20 and 21 [44]) which are known to confer resistance to EGFR Tyrosine Kinase Inhibitors (TKIs). Interestingly and of relevance for these TKI-resistant tumors, pre-exposure of H1975 cells to OVs appeared to sensitize the tumor cells to EGFRxCD16 BsAb-specific killing. Additionally, as mentioned above, although the production of GM-CSF should not directly impact NK cytotoxic function, the GM-CSF transgene inserted in the E3 region in oncolytic (adeno-)viruses was reported to enhance viral replication via autocrine JAK2/STAT2 signaling [45]. Given this superior viral fitness and consequent sensitization, one can also speculate that higher levels of stress molecules (i.e., MICA/B and ecto-calreticulin [10, 43]) may be expressed in ONCOS-102-infected tumor cells that may indirectly augment NK cytotoxicity.

The study focuses on the combinatorial effect of OVs and an EGFRxCD16 engager on NK cells, but the BsAb could also potentially trigger the activity of non-NK CD16 + cells, such as non-classical/intermediate monocytes and γδ T cells. Furthermore, because EGFR blockade with monoclonal antibodies like cetuximab can sensitize cancer cells to chemotherapy [46] or radiotherapy [47], one could wonder whether EGFRxCD16 BsAb—via its EGFR interaction—could similarly sensitize the tumor cells to the antitumor activity of CD16 + effector cells. Moreover, while GM-CSF and ICOS-L expression may not directly modulate NK cells, their indirect impacts mediated through other immune cells were not explored in this study. Further investigations, using more complex systems and readouts (i.e., in vivo and scRNAseq), will be instrumental in further refining and fully realizing the potential of the combination of OVs and EGFRxCD16 BsAb.

In conclusion, our study highlights that combining OVs tumor preconditioning with EGFRxCD16 BsAb, results in an additive NK cells antitumor effect. This enhancement occurs regardless of EGFR expression or mutation status, particularly in ovarian and EGFR-mutant NSCLC cell lines. Oncolytic virotherapy alone has yet to achieve widespread clinical success: A recent trial (NCT02963831) where ONCOS-102 OVs delivered in combination with durvalumab (anti-PD-L1) failed to control the disease thresholds in patients with ovarian cancer. Our findings underscore that the choice of immunomodulatory association may be a decisive factor in overcoming tumor-specific resistance and provide an alternative combinatorial approach.

## Electronic supplementary material

Below is the link to the electronic supplementary material.

Supplementary file 1 (DOCX 1020 kb)

## Data Availability

All data supporting the current study are presented within the article. Data related to this study may be requested from the corresponding author.
