# Supplementary material for: EGFRxCD16 bispecific antibodies orchestrate superior NK cell-mediated lysis of ovarian cancer and NSCLC cell lines in combination with oncolytic viruses
Source: Cancer Immunol Immunother. 2026 Jul 3;75(7):178. doi: 10.1007/s00262-026-04470-4 (PMC13332092; doi:10.1007/s00262-026-04470-4)

**Supplementary Tables**

**Suppl Table 1A: List of antibodies for phenotype analysis NK cells**

| Antibodies | Dye | clone | company | cat num |
| --- | --- | --- | --- | --- |
| NKp46 | AF488 | 9E2 | Thermo Fisher | 53-3359-42 |
| CX3CR1 | PE | K0124E1 | BioLegend | 355704 |
| CD57 | PE-CF594 | NK-1 | BD | 562488 |
| CD3 | PE-Cy7 | HIT3a | BioLegend | 300316 |
| TIGIT | APC | VSTM3 | BioLegend | 372706 |
| CD56 | AF700 | B159 | BD | 557919 |
| CD38 | APC-Fire 810 | HB-7 | BioLegend | 356643 |
| CD161 | BV421 | DX12 | BD | 562615 |
| Live/Dead marker | DCM aqua |  | Thermo Fisher | L34957 |
| NKG2D | BV650 | 1D11 | BD | 563408 |
| CD16 | BV785 | 3G8 | BioLegend | 302046 |

**Suppl Table 1B: List of antibodies for functional analysis of NK cells**

| Ab | Dye | clone | company | cat num |
| --- | --- | --- | --- | --- |
| Eomes | FITC | WD1928 | Thermo Fisher | 11-4877-42 |
| CD107a | PE |  | BD | 555801 |
| TNF | PE/eFluor610 | Mab11 | Thermo Fisher | 61-7349-42 |
| CD3 | PerCP-Cy5.5 | UCHT1 | BioLegend | 300430 |
| FasL | PC7 | NOK-1 | BioLegend | 306418 |
| IFNγ | APC | 4S.b3 | BD | 551385 |
| CD56 | A700 | B159 | BD | 557919 |
| T-bet | BV421 | O4-46 | BD | 563318 |
| Live/Dead marker | DCM aqua |  | Thermo Fisher | L34957 |
| CD69 | BV650 | FN50 | BioLegend | 310934 |

**Suppl Figure 1: Validation of EGFR-mutated cell line H1975. a.** Production of GM-CSF and surface expression of ICOS-L by H1975 tumor cells induced by different MOI (0, 10, 100, and 1000) of ONCOS-102 and ONCOS-204, respectively. **b.** Histogram representing the lack of binding of EGFRxCD16 BsAb on EGFR- Kasumi-1 tumor cell line. **c.** Titration of EGFRxCD16 BsAb on NK cells by absolute expression of CD107a **d.** Validation of NK activation against H1975 cells with EGFRxCD16 BsAb. Gating strategy for (**e**) phenotype and (**f**) functional analyses.

**Suppl Figure 2: Minor changes in NK cells phenotype induced by EGFRxCD16 BsAb. a.** Representative flow cytometry of CD57, CD161, NKp46, and TIGIT expression in CD3-CD56+ NK cells. Analysis of changes in frequency of CD57, CD161, NKp46, and TIGIT in NK cells induced by EGFRxCD16 BsAb after 24h co-culture with (**b**) A549 cells and (**c**) SK-OV-3 cells. Phenotypic changes induced by EGFRxCD16 BsAb after 24h co-culture with H1975 cells (**d**) and A375 cells (**e**). Analysis of changes in frequency of CD57, CD161, NKp46, and TIGIT in CD56dim NK cells induced by EGFRxCD16 BsAb after 24h co-culture with (**f**) A549 cells and (**g**) SK-OV-3 cells. N=4–6 individuals. The Wilcoxon test was used to compare the two conditions and subsets. * p<0.05.

**Suppl Figure 3: EGFRxCD16 BsAb increased NK cell function.** Increased functionality of NK cells induced by EGFRxCD16 BsAb after 4h co-culture with (**a**) A549 cells and (**b**) SK-OV-3 cells. NK cell transcription factor changes induced by EGFRxCD16 BsAb after 4h co-culture with (**c**) A549 cells and (**d**) SK-OV-3 cells. Different Eomes and Tbet co-expression in CD3-CD56+ NK cells after co-culture with BsAb and (**e**) A549 or (**f**) SK-OV-3 cells. N=4–6 individuals. The Wilcoxon test was used to compare the two conditions. * p<0.05.

**Suppl Figure 4: OV-infected tumor cells induced minor phenotypic changes in NK cells.** Analysis of phenotypic changes in CD56bright NK cells induced by 24h co-culture with OV-infected (**a & d**) A549 and (**b & e**) SK-OV-3 cells. **c.** Analysis of changes in CX3CR1 expression in CD56bright NK cells induced by different OV-infected tumor cells. Analysis of phenotypic changes in NK cells induced by 24h co-culture with OV-infected (**f-g**) H1975 and (**h-i**) A375 cells. N=4-6 individuals. The Friedman test with Dunn’s correction was used to compare between the four different conditions. * p<0.05.

**Suppl Figure 5: OV-infected tumor cells alone induce minor changes in NK function.** Comparison of function (**a-b**) and transcription factors (**c-d**) of NK cells exposed to different OV-infected (**a-c**) H1975 and (**b-d**) A375 cells. N=4–5 individuals. The Friedman test with Dunn’s correction was used to compare between the four different conditions.

**Suppl Figure 6: Addition of EGFRxCD16 BsAb to co-culture of OV-infected tumor cells causes minor functional changes in NK cells.** Analysis of phenotypic changes in NK cells induced by EGFRxCD16 BsAb during 24h co-culture with OV-infected (**a**) H1975 and (**b**) A375 cells. Functionality of NK cells induced by EGFRxCD16 BsAb after 4h co-culture with OV-infected (**c-d**) tumor cells. Transcription factor expression after 4h co-culture with EGFRxCD16 BsAb and OV-infected (**e-f**) tumor cells. N=4–5 individuals. The Friedman test with Dunn’s correction was used to compare between the four different conditions. * p<0.05.

**Suppl Figure 7: Oncolytic ability and NK cytotoxic activity. a.** Co-culture of sorted NK cells with tumor cells supplemented with 250ng/ml EGFRxCD16 BsAb-induced tumor killing demonstrated by reduced absorbance using WST-1 Cell Proliferation assays. **b**. Comparison of percentage of EGFRxCD16 BsAb-specific killing among different tumor cell lines. *Specific killing (%) = 100x(1-(Abs NK+BsAb)/(Abs NK alone)*. **c**. OVs-induced tumor killing demonstrated by absorbance difference using WST-1 Cell Proliferation assay. Comparison of percentage of OV-specific killing between different OVs and cell lines (**d**) without addition of EGFRxCD16 BsAb (Formula used: *specific killing (%) = 100x(1-(Abs NK+OVs)/(Abs NK alone)*) or (**e**) in combination with EGFRxCD16 BsAb (Formula used: *specific killing (%) = 100x(1-(Abs NK+BsAb+OVs)/(Abs NK+BsAb)*). N=6 individuals. The Wilcoxon test was used to compare the two conditions and subsets (**a**), the Kruskal–Wallis test was used to compare killing between tumor cell lines (**b**), and the Friedman test with Dunn’s correction was used to compare between the four different conditions (**c-e**). * p<0.05 and ** p<0.01. Abs: absorbance.


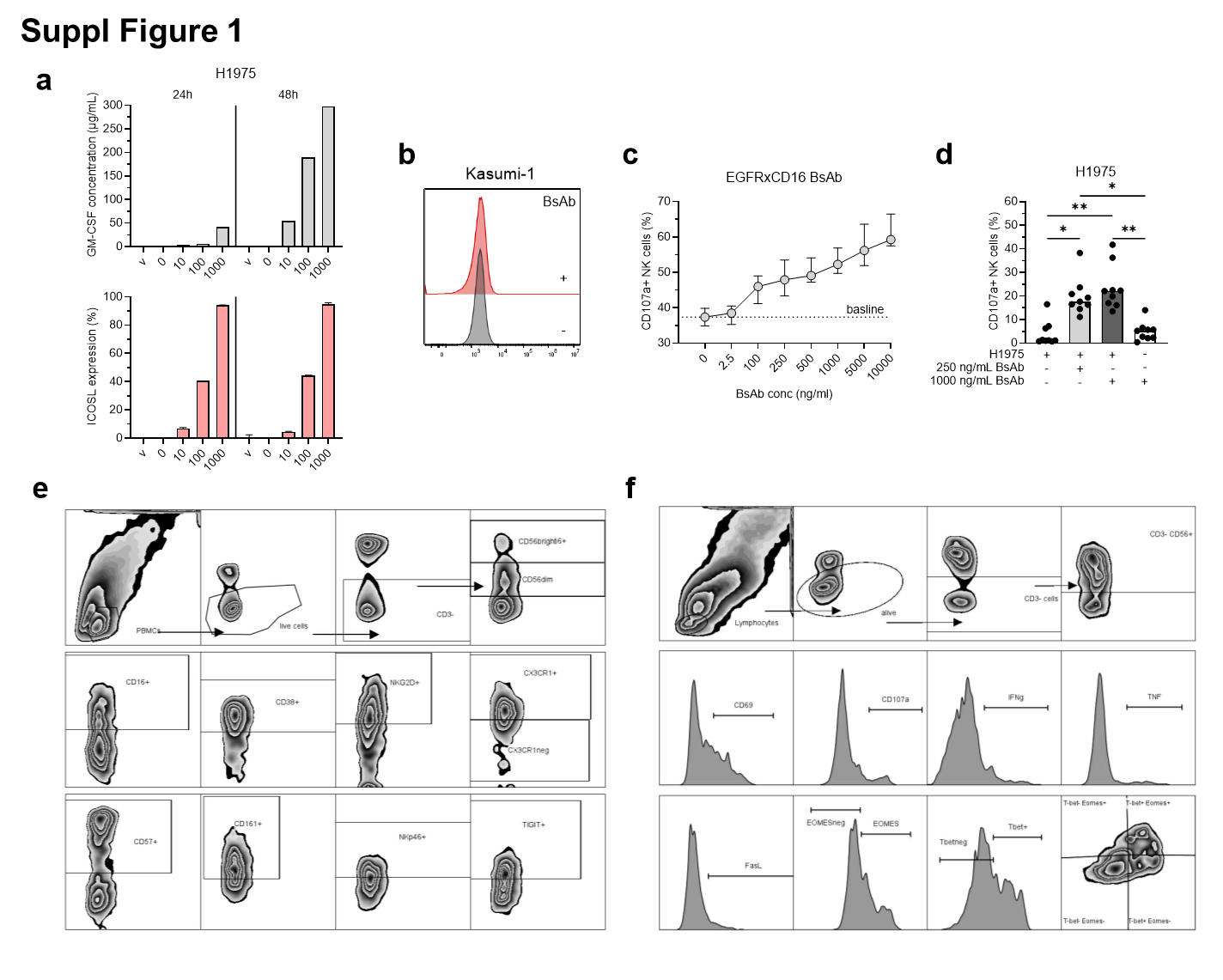


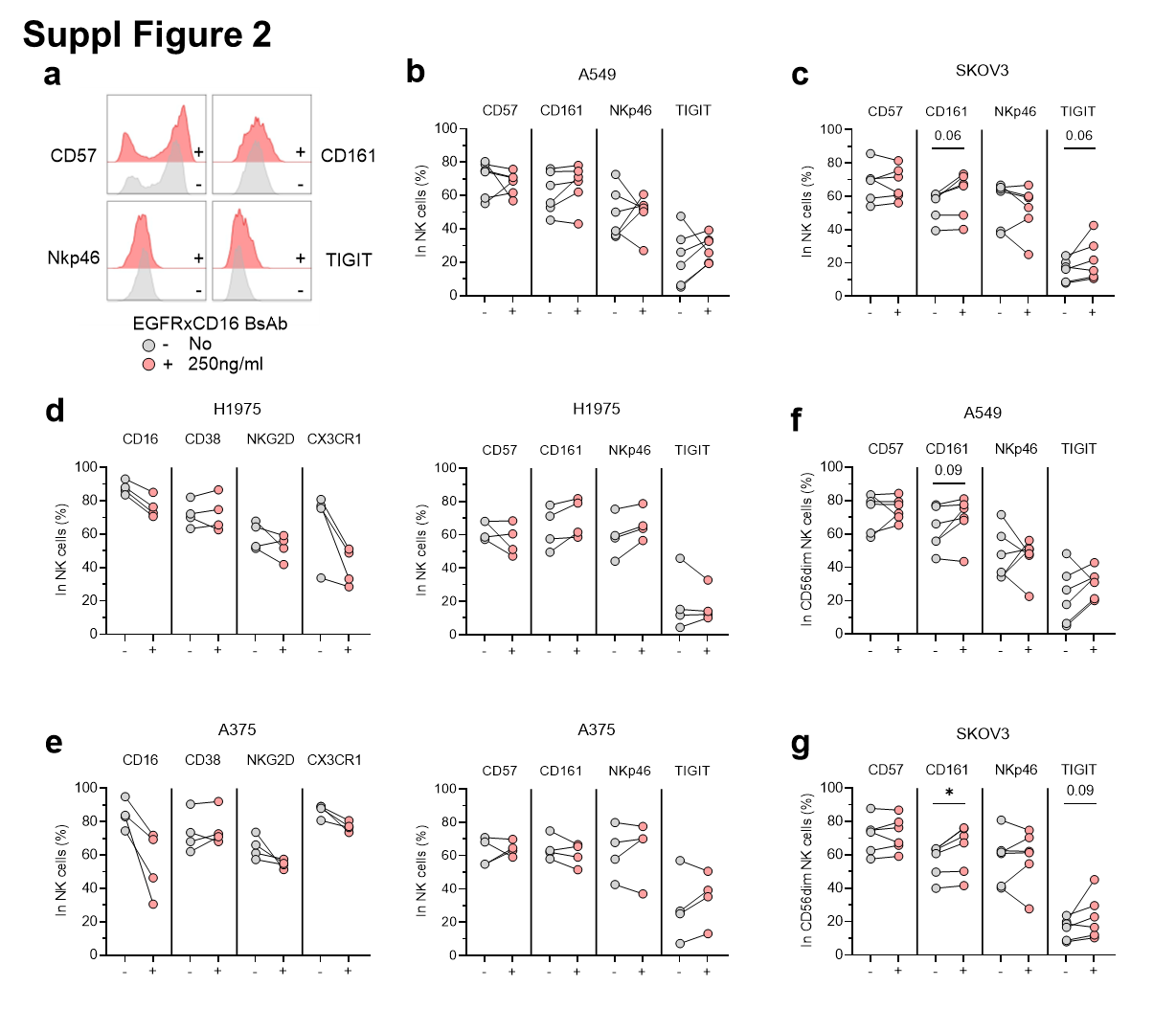


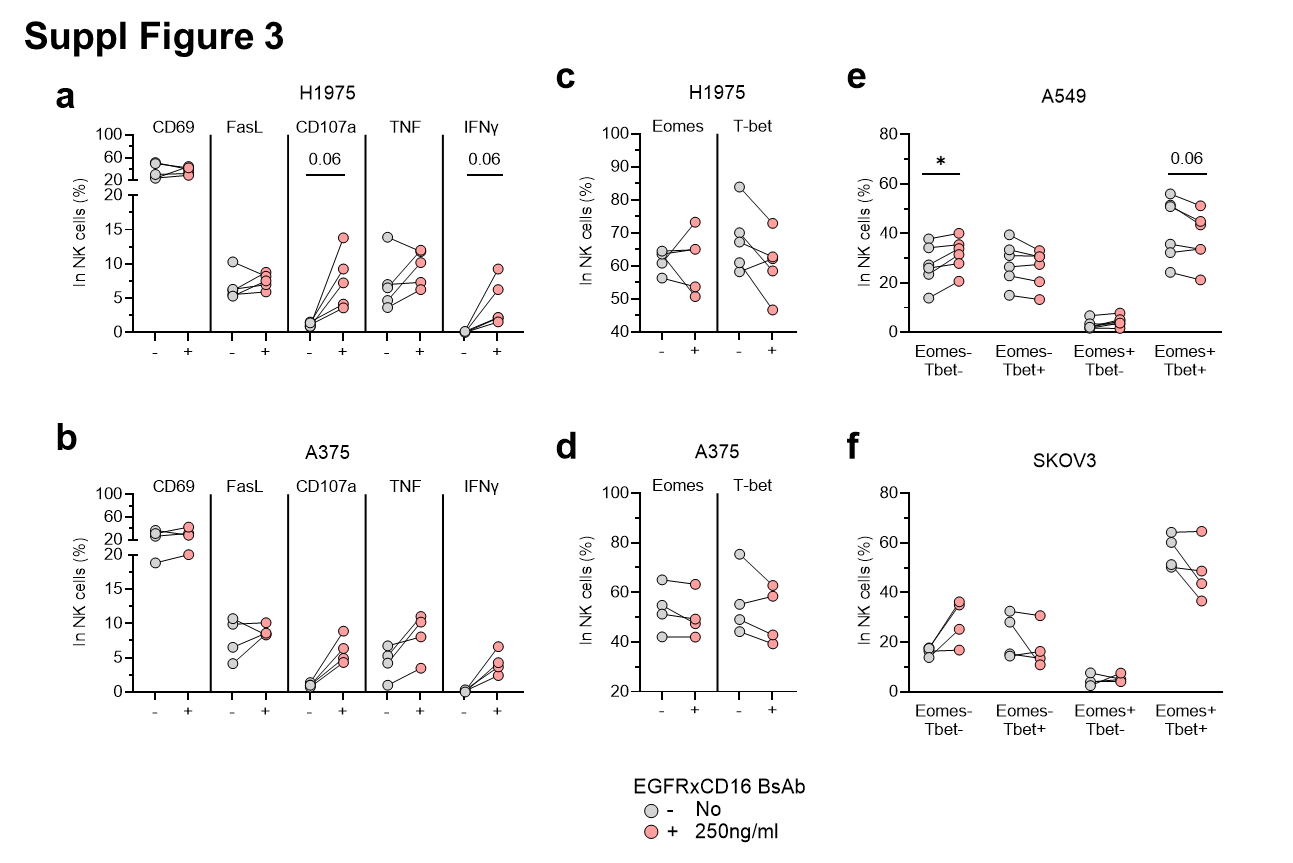


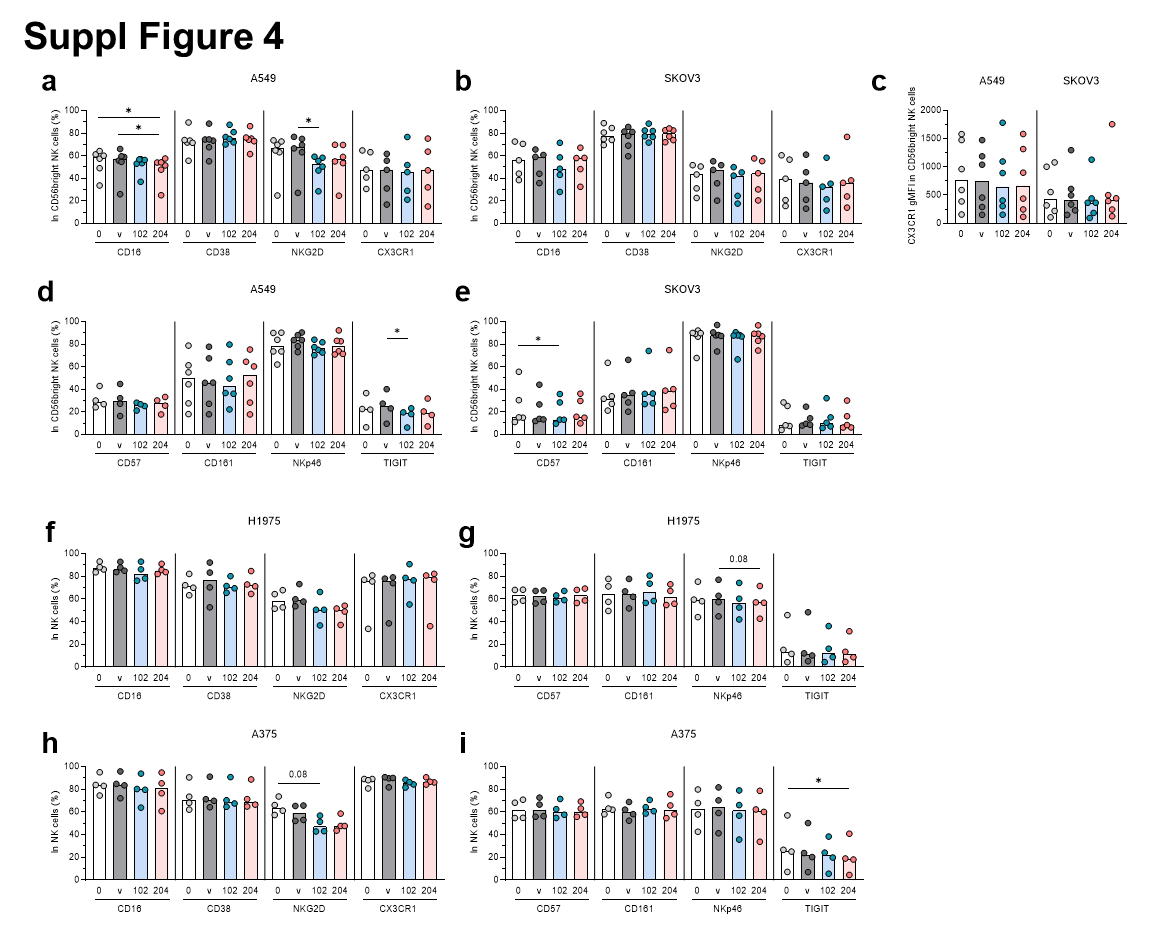


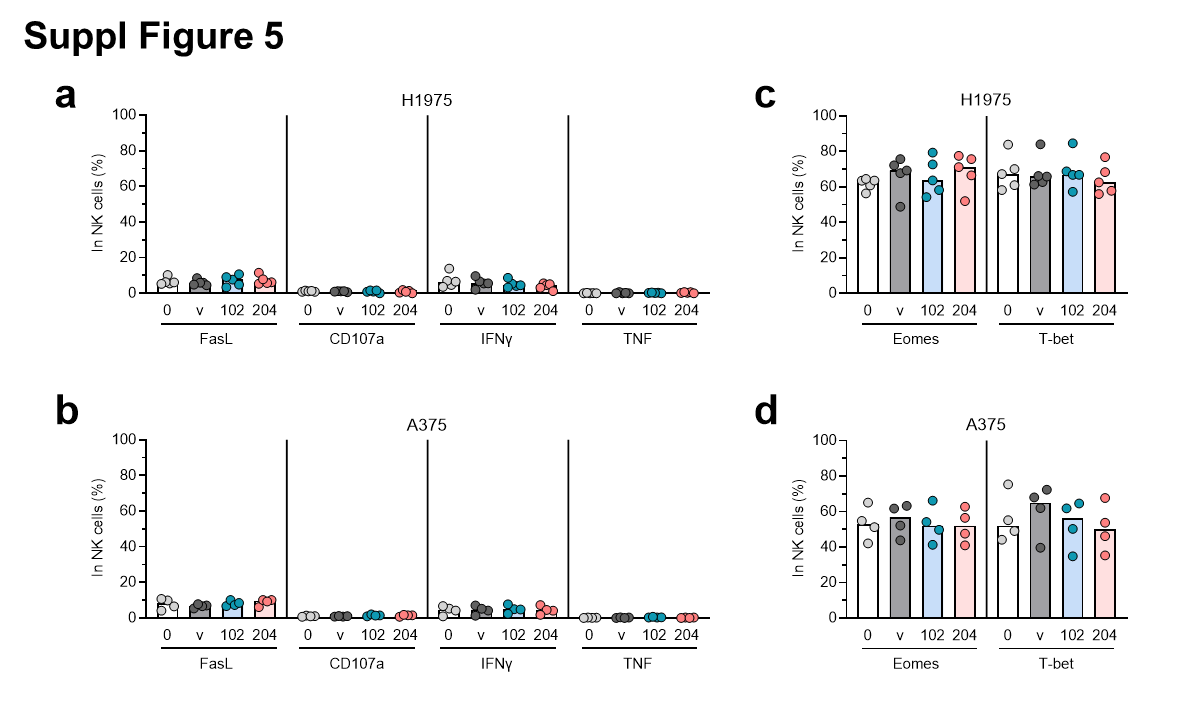


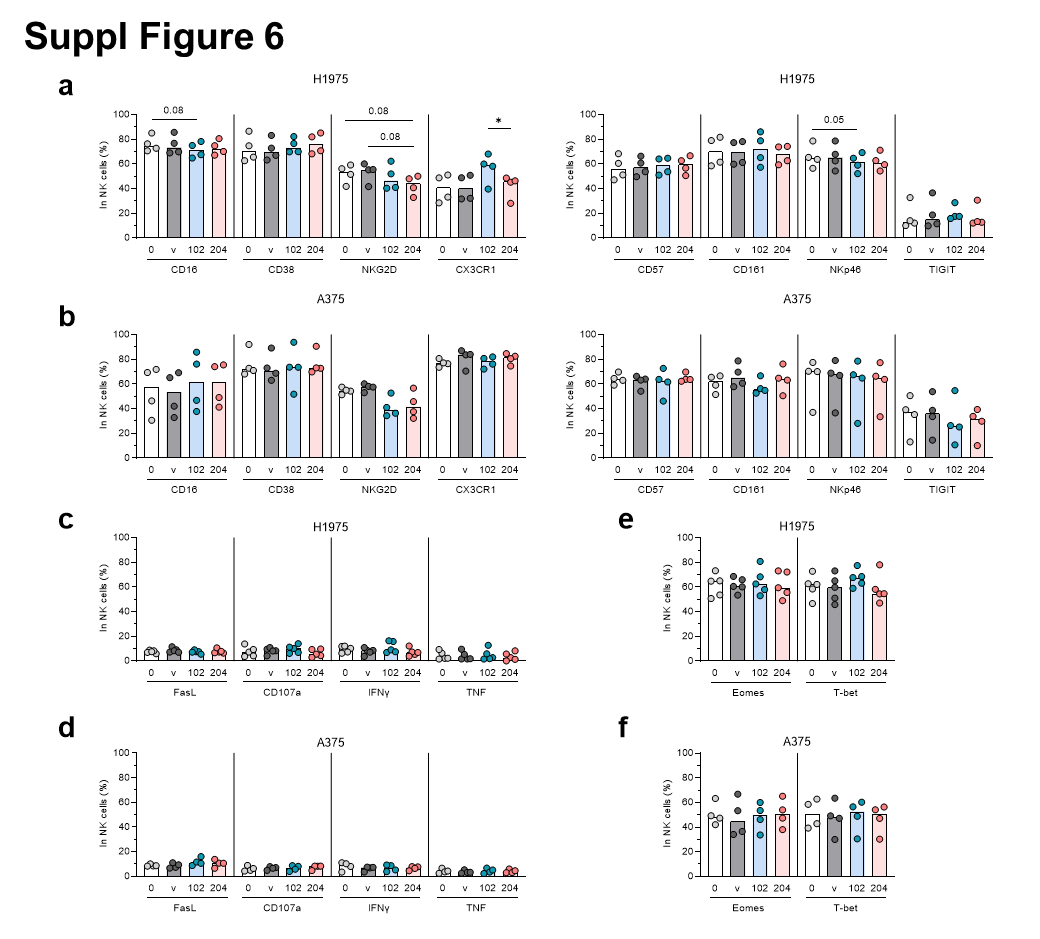


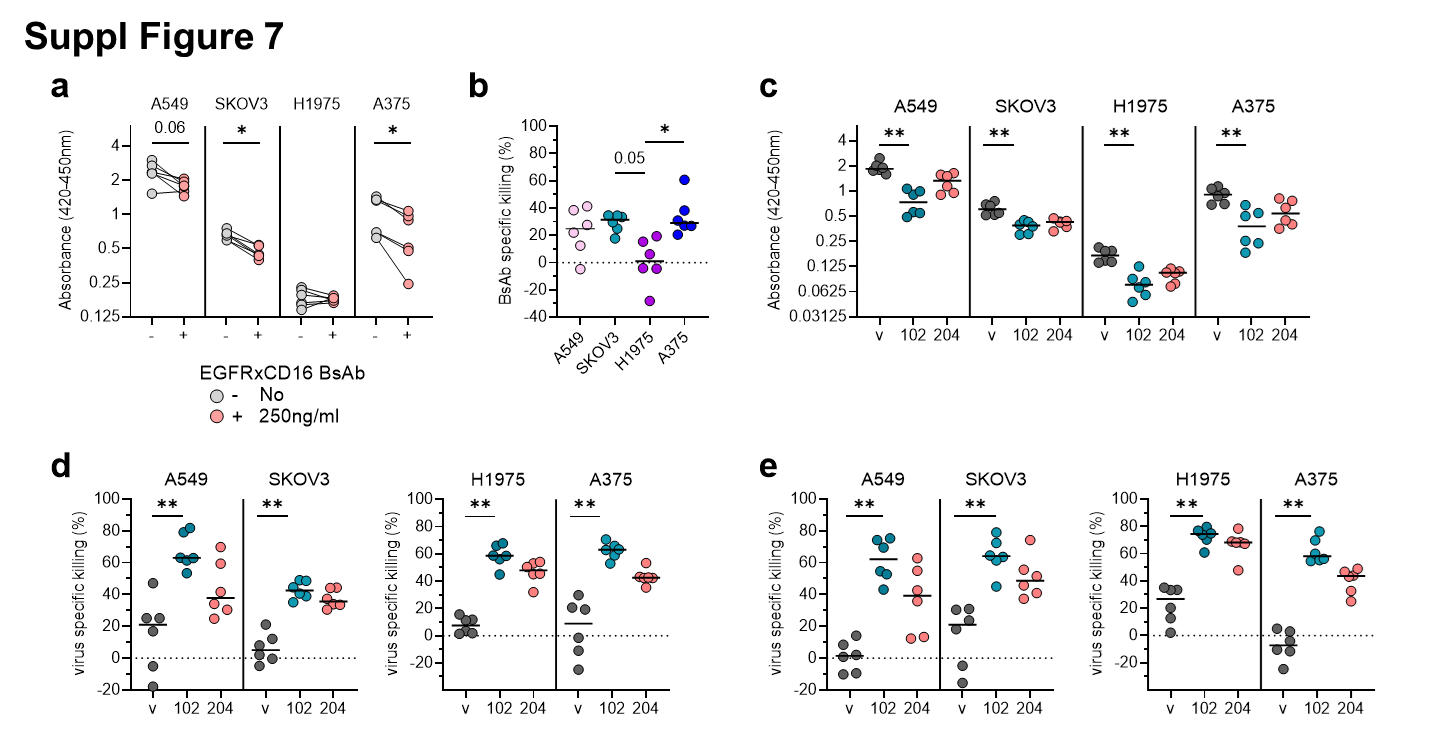

Supplement: Supplementary file 1 — (DOCX 1020 kb) [file 262_2026_4470_MOESM1_ESM.docx]
